# Investigation of perfusion impairment in degenerative cervical myelopathy beyond the site of cord compression

**DOI:** 10.1038/s41598-023-49896-3

**Published:** 2023-12-19

**Authors:** Anna Lebret, Simon Lévy, Nikolai Pfender, Mazda Farshad, Franziska C. S. Altorfer, Virginie Callot, Armin Curt, Patrick Freund, Maryam Seif

**Affiliations:** 1 Spinal Cord Injury Center, Balgrist University Hospital, Zürich, Switzerland; 2 CNRS, CRMBM, Aix-Marseille University, Marseille, France; 3 APHM, CEMEREM, Hôpital Universitaire Timone, Marseille, France; 4 MR Research Collaborations, Siemens Healthcare Pty Ltd, Melbourne, Australia; 5 Department of Orthopedic Surgery, Balgrist University Hospital, Zürich, Switzerland; 6 Department of Brain Repair and Rehabilitation, Wellcome Trust Center for Neuroimaging, Institute of Neurology, University College London, London, UK; 7 Department of Neurophysics, Max Planck Institute for Human Cognitive and Brain Sciences, Leipzig, Germany

**Keywords:** Neurodegeneration, Neurodegenerative diseases, Spinal cord diseases, Imaging techniques

## Abstract

The aim of this study was to determine tissue-specific blood perfusion impairment of the cervical cord above the compression site in patients with degenerative cervical myelopathy (DCM) using intravoxel incoherent motion (IVIM) imaging. A quantitative MRI protocol, including structural and IVIM imaging, was conducted in healthy controls and patients. In patients, T2-weighted scans were acquired to quantify intramedullary signal changes, the maximal canal compromise, and the maximal cord compression. T2*-weighted MRI and IVIM were applied in all participants in the cervical cord (covering C1–C3 levels) to determine white matter (WM) and grey matter (GM) cross-sectional areas (as a marker of atrophy), and tissue-specific perfusion indices, respectively. IVIM imaging resulted in microvascular volume fraction ($F$), blood velocity ($D^{*}$), and blood flow ($F \cdot D^{*}$) indices. DCM patients additionally underwent a standard neurological clinical assessment. Regression analysis assessed associations between perfusion parameters, clinical outcome measures, and remote spinal cord atrophy. Twenty-nine DCM patients and 30 healthy controls were enrolled in the study. At the level of stenosis, 11 patients showed focal radiological evidence of cervical myelopathy. Above the stenosis level, cord atrophy was observed in the WM (− 9.3%; *p* = 0.005) and GM (− 6.3%; *p* = 0.008) in patients compared to healthy controls. Blood velocity (BV) and blood flow (BF) indices were decreased in the ventral horns of the GM (BV: − 20.1%, *p* = 0.0009; BF: − 28.2%, *p* = 0.0008), in the ventral funiculi (BV: − 18.2%, *p* = 0.01; BF: − 21.5%, *p* = 0.04) and lateral funiculi (BV: − 8.5%, *p* = 0.03; BF: − 16.5%, *p* = 0.03) of the WM, across C1–C3 levels. A decrease in microvascular volume fraction was associated with GM atrophy (*R* = 0.46, *p* = 0.02). This study demonstrates tissue-specific cervical perfusion impairment rostral to the compression site in DCM patients. IVIM indices are sensitive to remote perfusion changes in the cervical cord in DCM and may serve as neuroimaging biomarkers of hemodynamic impairment in future studies. The association between perfusion impairment and cervical cord atrophy indicates that changes in hemodynamics caused by compression may contribute to the neurodegenerative processes in DCM.

## Introduction

Degenerative cervical myelopathy (DCM) is the most common cause of non-traumatic spinal cord injury and affects mostly the elderly population^1^. DCM is caused by progressive stenosis of the cervical spinal canal due to spinal osteoarthritic changes. The stenosis leads to compression of the cervical cord in DCM^2^ and initiates a series of primary and secondary injuries, such as apoptosis, microvascular deficits, and irreversible degeneration of axonal fibres, at and remote from the site of compression in the spinal cord^3–7^ and brain^8^. To prevent irreversible tissue damage and identify tissue at risk in the early stages of DCM, it is essential to understand the underlying pathophysiological processes contributing to spinal cord neurodegeneration, not only at the site of cord compression, but also in the non-compressed segments. Preclinical evidence indicates that perfusion impairment and vascular changes in the cervical cord play an important role in the development of DCM^9–12^, where perfusion deficit may be a potential precursor of neuronal cell death, leading eventually to tissue degeneration^13^.

Perfusion in DCM has been investigated using invasive contrast agent-based methods, mainly at the compressed level^14,15^. While remote neurodegeneration is evident above and below the compression level^5–7^, remote hemodynamic impairment and perfusion alteration of the cervical cord in DCM patients are still understudied due to the lack of non-invasive methods sensitive to blood perfusion. Thus, there is a pressing need for non-invasive techniques to investigate perfusion changes as a potential biomarker of tissue damage in DCM. Intravoxel incoherent motion (IVIM) imaging is a non-invasive, diffusion-based quantitative MRI method, indirectly sensitive to tissue microcirculation and perfusion^16^ which has been successfully applied in different brain pathologies^17–19^, and in the healthy cervical cord^20^.

This study therefore aimed to utilize IVIM imaging to investigate remote hemodynamic changes that could parallel neurogenerative changes in the cervical cord of DCM patients. A quantitative MRI protocol containing high-resolution structural scans and IVIM imaging was performed in the cervical cord (C1-C3 levels) of patients with mild-moderate DCM and their corresponding healthy controls to evaluate remote tissue-specific perfusion changes rostral to the compression site. We hypothesized that perfusion deficit occurs in a tissue-specific distribution, mainly in the parts of the cervical cord undergoing DCM-induced neurodegeneration (e.g., corticospinal and spinothalamic tracts, and dorsal columns^21^), rostral to the compression level. We furthermore aimed to determine the relation between remote perfusion impairment and the degree of macrostructural degeneration (e.g., atrophy), as well as with the functional and neurologic deficits in DCM.

## Methods

### Participants and study design

Twenty-nine DCM patients (mean age ± SD: 56.9 ± 11.3 years, 11 females), with mild (modified Japanese Orthopaedic Association [mJOA]: 15 ≤ mJOA ≤ 18) and moderate (12 ≤ mJOA ≤ 14) DCM, and 30 healthy controls (mean age ± SD: 55.4 ± 12.1 years, 16 females) were recruited at the Balgrist University Hospital (Spinal Cord Injury Center outpatient clinic) between June 2021 and December 2022. The inclusion criteria included a diagnosis of DCM, radiological evidence of spinal cord stenosis on the structural MRI scans, no prior operation to the cervical spine, no pregnancy, no preexisting neurological condition, and age between 18 and 75 years old. One healthy control was excluded from the analysis due to the presence of preexisting spinal cord compression.

### Standard protocol approvals, registrations, and patient consents

The study protocol was designed according to the Declaration of Helsinki and approved by the local Ethics Committee (Kantonale Ethikkommission Zürich, EK-2018–00,937). Written informed consent was obtained from each participant before study enrolment.

### Clinical assessment

A clinical examination protocol was performed on DCM patients to assess functional and neurologic deficits, including the evaluation of the mJOA classification, to score the patient functional status by quantifying the level of clinical deficit in the upper and lower limbs (max. 18 points)^22^, and of the Nurick grade to classify the walking ability of the patient on a 5-point scale^23^. Functional and sensory hand deficits were assessed by performing the prehension and sensibility test as a subset of the Graded and Redefined Assessment of Strength, Sensibility and Prehension (GRASSP) test, which has score of maximum 132 points. Moreover, the International Standards for Neurological Classification of Spinal Cord Injury protocol (ISNCSCI)^24^ was carried out, including tests of the upper extremity motor score (UEMS) (max. 50 points), cervical light-touch (CLT) and cervical pinprick (CPP) (across C2-C8 spinal segments) (max. 28 points). Three patients were not assessed clinically due to lack of time.

### Image acquisition

All MRI data were acquired on a 3T MRI scanner (MAGNETOM Prisma, Siemens Healthcare, Erlangen, Germany) with a Siemens Healthcare 64-channel head and neck radio-frequency coil. To minimize motion artifacts in the inferior-superior direction, all participants were asked to wear a stiff neck (Laerdal Medicals, Norway). Participants were placed head-first supine in the scanner. The participants were instructed to avoid swallowing during the scans to reduce tissue motion. All scans were acquired at the cervical cord level covering C1-C3 levels. The nominal total acquisition time was 41 min.

### Anatomical MRI measurements

To calculate the maximum spinal cord compression and canal compromise and for the localization of the cervical segments, a sagittal T2-weighted turbo spin echo (TSE) sequence was acquired with the following imaging parameters: repetition time (TR): 3500 ms, echo time (TE): 84 ms, flip angle: 160°, field of view (FOV): 220 × 220 mm^2^, in-plane resolution: 0.3 × 0.3 mm^2^, 20 slices, slice thickness: 2.5 mm, readout bandwidth: 260 Hz/pixel, acquisition time: 1:47 min, using parallel imaging with an acceleration factor (iPAT) of 2 using a generalized autocalibration partially parallel acquisition algorithm (GRAPPA).

To evaluate the lesion site, an axial T2-weighted TSE sequence was additionally applied with the following MRI parameters: TR: 5510 ms, TE: 96 ms, flip angle: 150°, FOV: 160 × 160 mm^2^, in-plane resolution: 0.6 × 0.6 mm^2^, 23 slices, slice thickness: 3 mm, readout bandwidth: 283 Hz/pixel, acquisition time: 1:57 min, with GRAPPA and iPAT 2.

To quantify cross-sectional areas of the grey and white matter, an axial T2*-weighted 3D multi-echo gradient-echo scan was acquired, with MRI parameters as follows: TR: 38 ms, 5 echoes time: TE [6.85, 10.85, 14.85, 18.85, 22.85] ms, flip angle: 8°, FOV: 192 × 192 mm^2^, in-plane resolution: 0.5 × 0.5 mm^2^, 16 slices, slice thickness: 5 mm, readout bandwidth: 260 Hz/pixel, acquisition time: 6:29 min. To reduce the overall acquisition time, GRAPPA with an acceleration factor of 2 was used in the phase-encoding direction.

### IVIM MRI measurements

To assess perfusion, the vendor product 2D-RF diffusion-weighted spin-echo EPI ZOOMit (Zonally-magnified Oblique Multislice)^25^ sequence was used in transversal orientation with cardiac-gating, trigger delay of 100 ms, TR of 600 ms, nominal TE of 58 ms, 0.9 × 0.9 mm^2^ in-plane resolution, FOV of 101 × 31.8mm^2^, 9 slices with a 5 mm-slice thickness, 34 × 108 matrix size, bandwidth of 1402 Hz/pixel, and 3 concatenations. Fourteen b-values were acquired, ranging from 0 to 650 s/mm^2^ with an increment of 50 s/mm^2^, and with twenty repetitions per b-value in three in-plane diffusion encoding directions, based on the previous report on optimizing b-values and diffusion-encoding directions^26^. The acquisitions were split into forward and reverse phase-encoding directions for subsequent distortion correction. The nominal acquisition time per phase-encoding direction was 5:08 min (the actual acquisition time was dependent on the participant’s heartbeat and varied between 5 and 10 min).

### Image processing

#### Lesion segmentation and stenosis assessment based on T2-weighted MRI

The maximum spinal cord compression (MSCC) and maximum canal compromise (MCC) provide the ratios of the strongest reduction in diameter of the spinal cord, spinal canal respectively, against the non-compressed diameter references from above and below the compressed site. They were calculated based on the mid-sagittal slice of the T2-weighted scans^27,28^ using Jim 7.0 software (Xinapse systems, Aldwincle, UK)^7^. Next, manual segmentation of the hyperintensity signal on axial T2-weighted scans was conducted in FSLeyes (FMRIB, Oxford, United Kingdom) to produce the frequency map of the cervical T2-hyperintensity in DCM patients with radiological myelopathy evidence determining the frequency of the lesions^29^. The manual segmentations of the lesions were conducted in native space. The resulting individual lesion masks were normalized to the PAM50 template with a slice-wise nonlinear registration step and voxel-wise averaging across individuals was conducted. The voxel intensity on the frequency map indicates the frequency of a lesion located in that voxel (in percentage) for the cohort under study^7^. 

#### Cross-sectional areas based on T2*-weighted MRI

Cross-sectional areas of the grey and white matter were obtained from automatic segmentations (subsequently manually corrected) on the axial T2*-weighted images using *sct_deepseg_sc*^30^ and *sct_deepseg_gm*^31^ from the Spinal Cord Toolbox (SCT, version 5.7)^32^. The cross-sectional areas were computed and averaged across C1-C3 cervical levels. One patient was excluded from the macrostructural morphometric assessment due to compression level within the field-of-view that degraded the image quality (the grey and white matter could not be differentiated).

#### IVIM MRI processing

IVIM data were processed using a custom-made IVIM toolbox^20^ to generate perfusion maps. In brief, the following processing steps were performed: denoising^33^, Gibbs artefacts removal using the local subvoxel-shifts method^34,35^ (*DIPY*^36^), motion (*sct_dmri_moco*^32^), and distortion correction (*FSL topup*^37^). Next, voxel-wise fitting of the IVIM signal was carried out to generate perfusion maps based on a biexponential IVIM model^16^:

$$S\left( b \right) = S_{0} e^{ - bD} \left[ {F e^{{ - bD^{*} }} + 1 - F} \right]$$

where $F$ is the microvascular volume fraction, $D^{*}$ is the pseudo-diffusion coefficient, and $D$ is the diffusion coefficient.

The IVIM maps were registered to the PAM50 template^38^ and white matter atlas^39^, with an intermediate registration step on the T2*-weighted image. The perfusion-sensitive parameters $F$, $D^{*}$ (providing information on the blood volume and blood velocity^40^, respectively), $F \cdot D^{*}$ (indirectly sensitive to blood flow^40^), and the diffusion coefficient $D$ were extracted from the individual perfusion maps in subject space across C1-C3 levels, in the white and grey matter. Further atlas-based analysis was conducted to assess tissue-specific perfusion within substructures of interest of the white matter (ventral funiculi, lateral funiculi and dorsal columns) and of the grey matter (ventral horns and dorsal horns). All regions of interest (ROIs) were eroded at the cord periphery to minimize partial volume effect with the cerebrospinal fluid.

Due to incorrect positioning of the field-of-view of the IVIM scan, one patient was excluded from the subsequent macrostructural and perfusion analysis. Due to strong motion artefacts, one patient was removed from the perfusion analysis. One scanning session was not fully conducted and the patient was therefore excluded from subsequent analysis. After exclusion of images due to different artefacts, 26 datasets from DCM patients and 29 from healthy controls were included in the macrostructural and perfusion analyses.

### Statistical analysis

All statistical analysis was performed in R software (version 4.1.2, R Core Team^41^). Differences in age and sex distribution between DCM patients and healthy controls were tested with the Mann–Whitney *U* test and Fisher’s exact test, respectively. First, remote spinal cord macrostructural (grey matter and white matter cross-sectional areas) and IVIM parameters differences were assessed above the compression site (C1-C3 levels) between DCM patients and healthy controls. Cross-sectional areas were compared between both cohorts in the ROIs using two-sample independent *t*-tests. One-tailed two-sample independent *t*-tests were conducted to compare IVIM parameters between DCM patients and healthy controls, and between DCM patients with and without radiological evidence of myelopathy, with a significance level of *α* = 0.05. A two-way analysis of variance (ANOVA) was conducted to assess perfusion differences in the substructures of the white matter (ventral funiculi, lateral funiculi, dorsal columns), followed by post-hoc analysis for comparisons between DCM patients and healthy controls.

Relationships between IVIM parameters and macrostructural morphometric measures (ROIs cross-sectional areas, MSCC, MCC, and stenosis level) were explored using linear regression and Pearson’s correlation coefficient. Finally, linear regression was conducted to reveal relationships between IVIM parameters and clinical outcome measures, as well as between macrostructural measures and clinical outcome measures, with a significance level of *α* = 0.05.

## Results

### Clinical, radiologic and neurologic characteristics

Twenty-nine DCM patients and 30 healthy controls were enrolled in the study. Patient data are reported in Table 1. The age and sex distributions were not significantly different between both cohorts, as tested by the Mann–Whitney *U* test (W = 455, *p* = 0.3) and Fisher’s exact test (*p* = 0.2), respectively. Sensorimotor assessment scores averaged across DCM patients were the following: upper extremity motor score: 49.5 ± 1.3 (min. score: 45, max. score: 50), cervical light-touch: 27.0 ± 1.4 (min. score: 24, max. score: 28), cervical pinprick: 26.7 ± 1.9 (min. score: 20, max. score: 28), and GRASSP: 125.1 ± 8.1 (min. score: 108, max. score: 132). The mJOA score was on average 16 ± 1.6 (min. score: 12, max. score: 18) (including twenty-three mild (15 ≤ mJOA ≤ 18) and six moderate (12 ≤ mJOA ≤ 14) patients), and Nurick score 0.9 ± 0.7 (min. score: 0, max. score: 2). The maximum compression level was located at C3-C4 for 5 patients (17.2%), at C4-C5 for 8 patients (27.6%), at C5-C6 for 15 patients (51.7%), and at C6-C7 for 1 patient (3.5%). MSCC was 25.7 ± 10.4% and MCC 39.5 ± 10.8%. Eleven DCM patients showed radiological evidence of cervical myelopathy (mean age ± SD = 62.1 ± 7.7 years, 2 females, mJOA = 15.3 ± 1.6), which was located at C3-C4 for 3 patients, at C4-C5 for 2 patients, and at C5-C6 for 6 patients.

**Table 1:** Clinical and neurological characteristics of DCM patients (n = 29).

Sex	N (%) or mean ± SD
Female	N = 11 (37.9%)
Male	N = 18 (62.1%)
Age [years]	56.9 ± 11.3
Clinical scores	
mJOA (0 – 18)	16 ± 1.6
Nurick (0 – 5)	0.9 ± 0.7
UEMS (0 – 50)	49.5 ± 1.3
CLT (0 – 28)	27.0 ± 1.4
CPP (0 – 28)	26.7 ± 1.9
GRASSP (0 – 132)	125.1 ± 8.1
Maximum stenosis level	
C3-C4	N = 5 (17.2%)
C4-C5	N = 8 (27.6%)
C5-C6	N = 15 (51.7%)
C6-C7	N = 1 (3.5%)
MSCC [%]	25.7 ± 10.4
MCC [%]	39.5 ± 10.8
Radiological evidence of myelopathy	N = 11 (37.9%)
Multi-segmental cervical spine stenosis	N = 21 (72.4%)

mJOA: modified Japanese Orthopedic Association index (maximum 18 points); Nurick (maximum 2 points); UEMS: Upper Extremity Motor Score (maximum 50 points); CLT: Cervical Light Touch (C2-C8, maximum 28 points); CPP: Cervical Pinprick (C2-C8, maximum 28 points); GRASSP: Graded and Redefined Assessment of Strength, Sensibility and Prehension (Sensibility and Prehension subset test; maximum 132 points); MSCC: Maximum Spinal Cord Compression; MCC: Maximum Canal Compromise.

**Figure 1:**
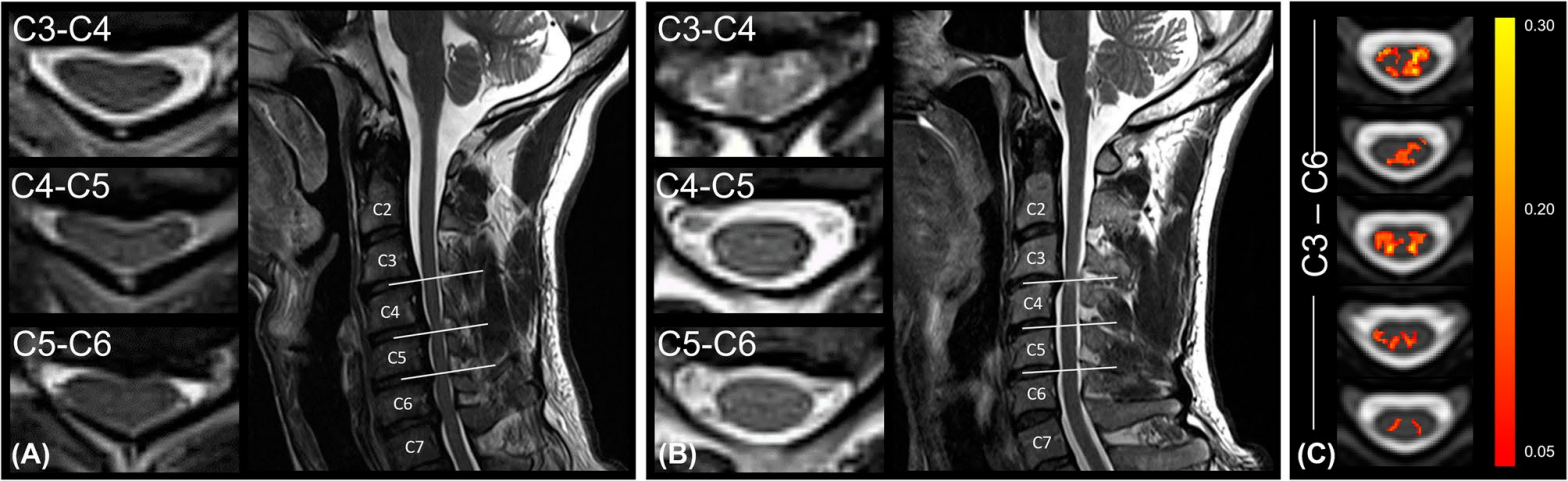
Lesion frequency map. Overview of the compression site in DCM patients (**A**) without and (**B**) with radiological hyperintensity myelopathy. The disk levels are indicated in the upper left corner. (**C**) Frequency map of radiological hyperintense signal along the cord of DCM patients with cervical myelopathy overlaid on the PAM50 template^38^.

The lesion frequency map indicated a higher frequency of radiological myelopathy in the grey matter at C3-C5 levels in the DCM group (Fig. 1).

### Remote macrostructural tissue-specific neurodegeneration

**Figure 2:**
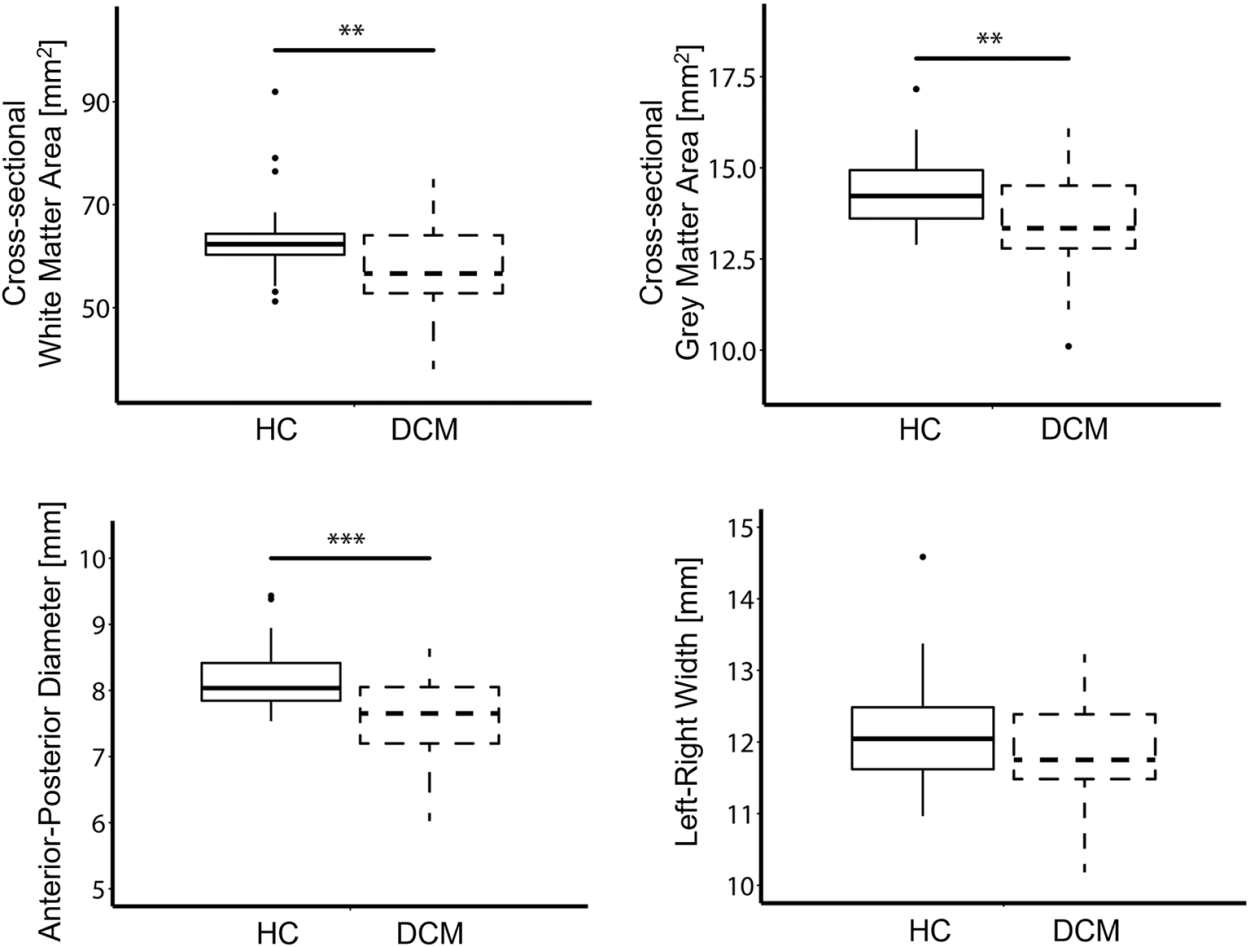
Remote macrostructural neurodegeneration. Box and whisker plots of the cross-sectional areas of white matter and grey matter, of spinal cord anterior–posterior diameter and left–right width in the cervical cord, averaged across C1-C3 levels, in healthy controls (HC) and DCM patients. **p < 0.01, ***p < 0.005.

Significant reduction in cross-sectional area above the compression site (suggesting neurodegeneration) was observed in DCM patients compared to healthy controls (HC) in the white matter (Δ =  − 9.3%; *p* = 0.005; HC: 63.5 ± 8.0 [mm^2^]; DCM: 57.6 ± 8.4 [mm^2^]) and grey matter (Δ =  − 6.3%; *p* = 0.008; HC: 14.4 ± 1.0 [mm^2^]; DCM: 13.5 ± 1.4 [mm^2^]) across C1-C3 levels. Similarly, the anterior–posterior diameter of the cervical cord was significantly lower in DCM patients than in healthy controls (Δ =  − 7.3%; *p* = 0.0005; HC: 8.2 ± 0.5 [mm]; DCM: 7.6 ± 0.6 [mm]), however there was no significant difference in left–right width between healthy controls and patients (HC: 12.1 ± 0.8 [mm]; DCM: 11.9 ± 0.8 [mm]) (Fig. 2).

### Remote hemodynamic impairment above the compression level

**Figure 3:**
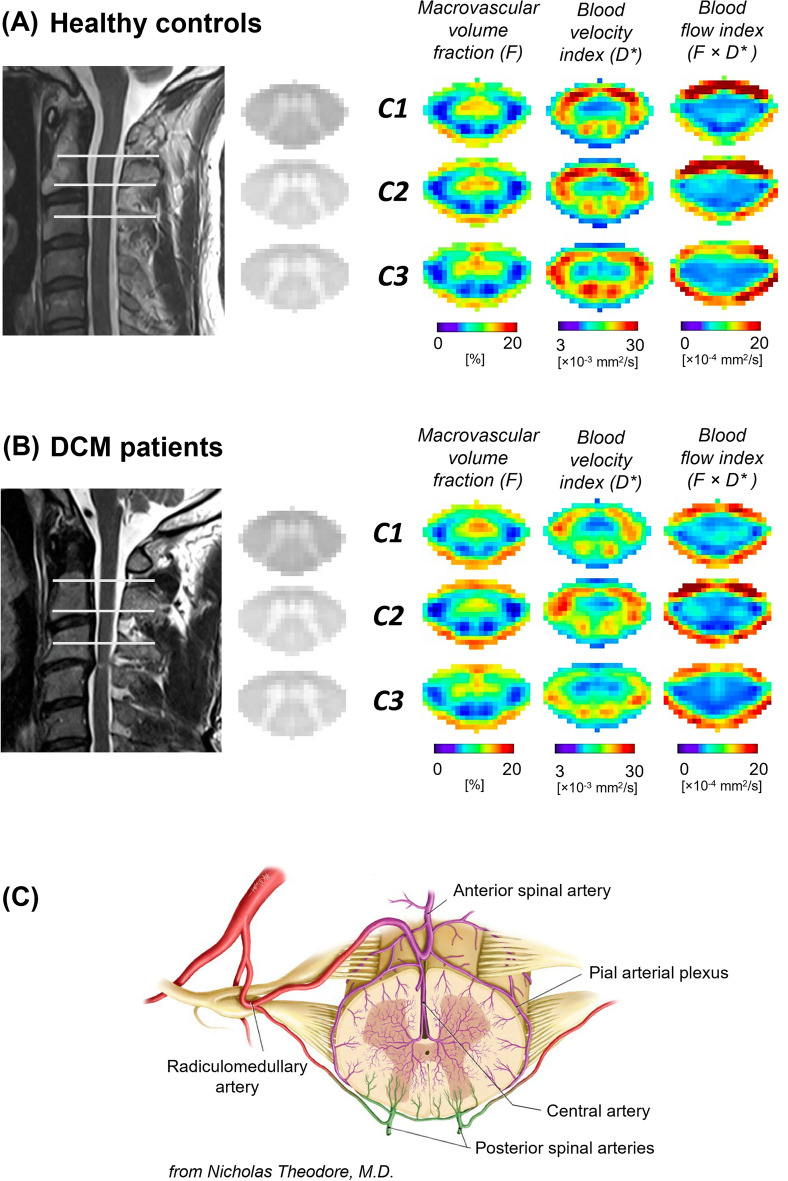
Perfusion maps in the cervical cord of healthy controls and patients with DCM. Sagittal T2-weighted image of a representative (**A**) healthy control and (**B**) DCM patient in subject space. High-resolution axial T2*-weighted MRI of the cervical cord in the template space at C1-C3 levels averaged across participants. IVIM mean maps including microvascular volume fraction (${\text{F}}$) [%], blood velocity index (${\text{D}}^{*}$) [mm^2^/s], and blood flow index (${\text{F}} \cdot {\text{D}}^{*}$) [mm^2^/s] in the template space (0.5 × 0.5 × 5 mm^3^) at C1-C3 levels. IVIM maps were averaged across diffusion-encoding directions and participants. (C) Vascularization of lumbar human spinal cord (used with permission from Nicholas Theodore, M.D.).

**Figure 4:**
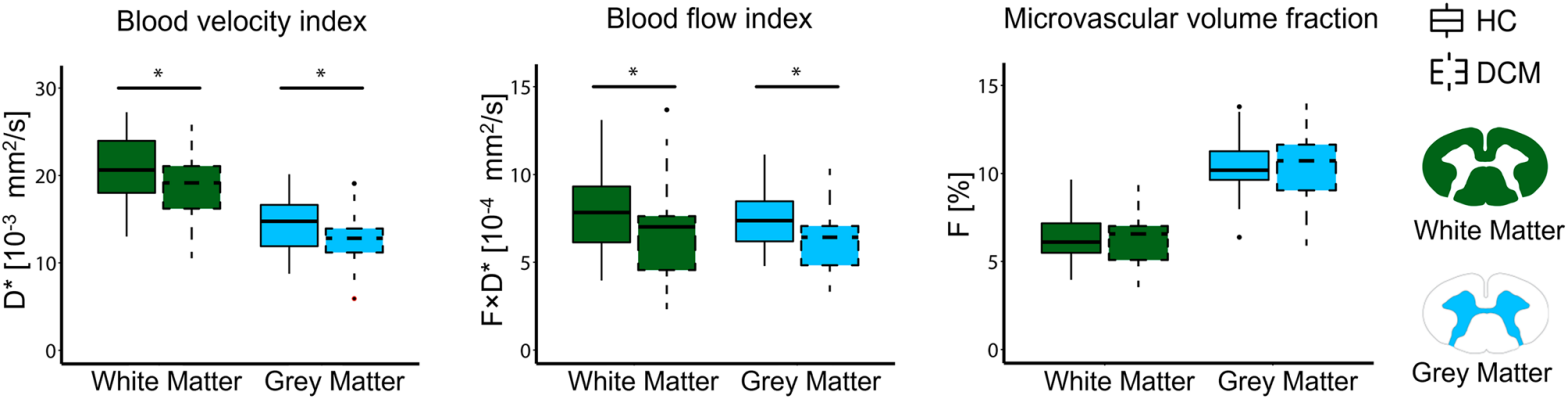
Perfusion differences between healthy controls and patients with DCM. Comparing IVIM parameters between healthy controls (HC) and DCM patients. Box and whisker plots of IVIM parameters: blood velocity index (${\text{D}}^{*}$) [mm^2^/s], blood flow index (${\text{F}} \cdot {\text{D}}^{*}$) [mm^2^/s] and microvascular volume fraction (${\text{F}}$) [%], averaged across slices over C1-C3 levels in HC and DCM groups. *p < 0.05.

Perfusion maps, averaged across participants, depicted the spatial distribution of the IVIM parameters in the cervical cord at C1-C3 levels (Fig. 3). While IVIM maps visually suggested a gradient of microvascular volume fraction ($F$) in the grey matter of DCM patients, decreasing from C1 to C3 (Fig. 3B), the value of the gradient was not statistically significant. Averaged values across cohorts of the IVIM parameters are reported in the white and grey matter in Supplementary Table S1. DCM patients showed a statistically significant decrease in the white matter compared to healthy controls in terms of blood velocity index ($D^{*}$) (Δ =  − 9.5%, *p* = 0.03, HC: 20.1 ± 3.9 × 10^−3^ [mm^2^/s], DCM: 18.7 ± 3.9 × 10^−3^ [mm^2^/s]) and blood flow index ($F \cdot D^{*}$) (Δ =  − 15.9%, *p* = 0.04, HC: 8.0 ± 2.5 × 10^−4^ [mm^2^/s], DCM: 6.7 ± 2.7 × 10^−4^ [mm^2^/s]) (Fig. 4). Additionally, in the grey matter, the blood velocity index was significantly reduced (Δ =  − 11.0%, *p* = 0.03, HC: 14.3 ± 3.1 × 10^−3^ [mm^2^/s], DCM: 12.7 ± 2.9 × 10^−3^ [mm^2^/s]), as was the blood flow index (Δ =  − 14.4%, *p* = 0.02, HC: 7.5 ± 1.6 × 10^−4^ [mm^2^/s], DCM: 6.4 ± 1.9 × 10^−4^ [mm^2^/s]). The microvascular volume fraction ($F$) did not significantly differ between healthy controls and DCM patients in the investigated ROIs (white matter: HC: 6.4 ± 1.5 [%], DCM: 6.3 ± 1.6 [%]; grey matter: HC: 10.4 ± 1.7 [%], DCM: 10.3 ± 2.1 [%]) (Fig. 4). No significant differences in IVIM perfusion indices were found between DCM patients with and without radiological evidence of myelopathy. No significant differences were observed in terms of diffusion coefficient ($D$) between healthy controls and DCM patients (white matter: HC: 4.0 ± 0.6 × 10^−4^ [mm^2^/s], DCM: 4.1 ± 0.6 × 10^−4^ [mm^2^/s]; grey matter: HC: 4.3 ± 0.6 × 10^−4^ [mm^2^/s], DCM: 4.4 ± 0.6 × 10^−4^ [mm^2^/s]).

**Figure 5:**
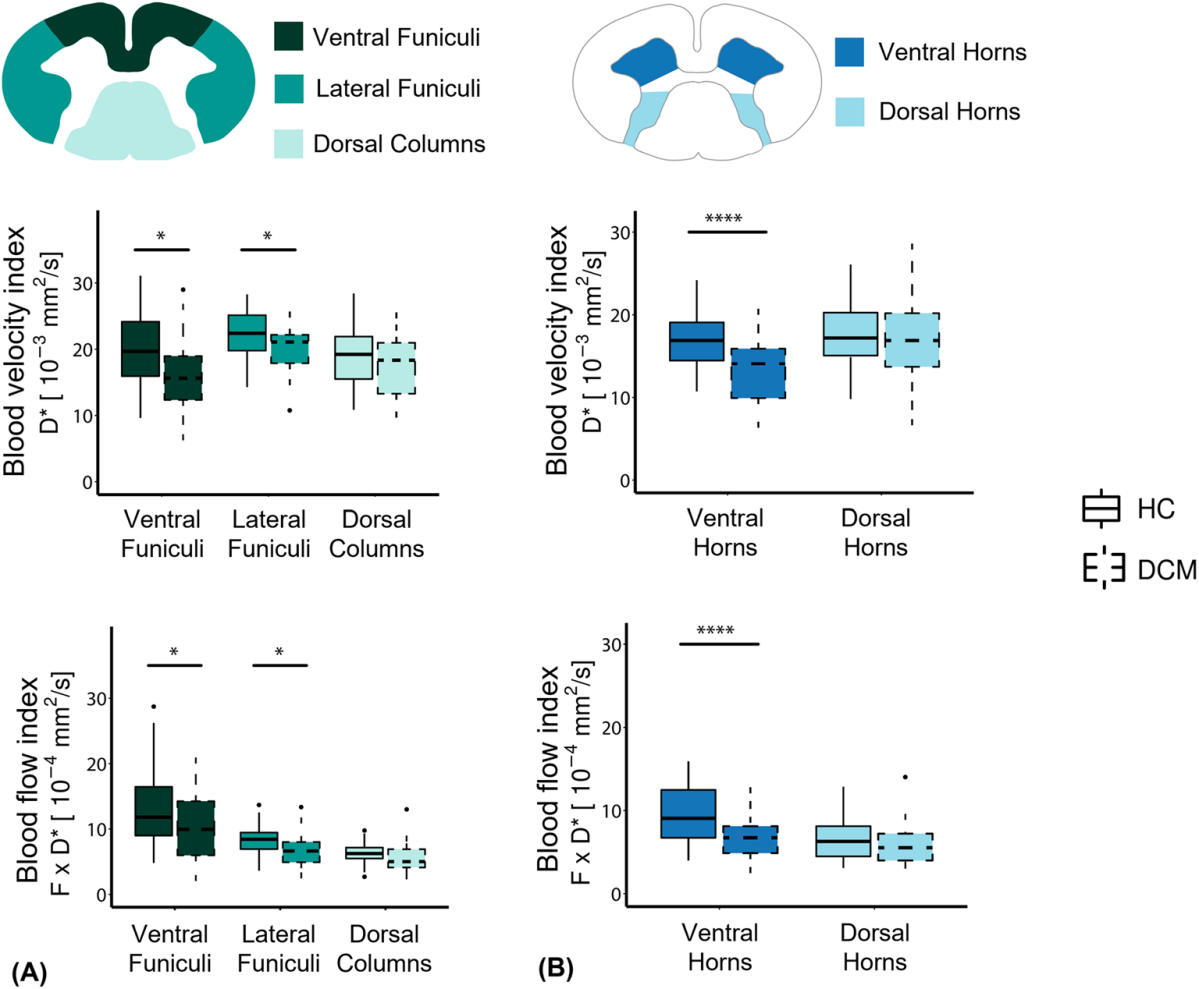
Tissue-specific hemodynamic impairment. Box and whisker plots of IVIM parameters showing values in white matter tracts and grey matter ventral and dorsal horns, in healthy controls (HC) and DCM patients. Blood velocity index (${\text{D}}^{*}$) [mm^2^/s] and blood flow index (${\text{F}} \cdot {\text{D}}^{*}$) [mm^2^/s] averaged across slices over C1-C3 levels in HC and DCM groups (**A**) in the ventral funiculi, lateral funiculi, and dorsal columns of the white matter and (**B**) in the ventral and dorsal horns of the grey matter. At the top, schematic representations of regions of interest in the white matter (ventral funiculi, lateral funiculi, dorsal columns) and grey matter (ventral horns, dorsal horns) are shown. *p < 0.05, ****p < 0.001.

Perfusion analysis in different tracts of the white matter and grey matter horns suggested impairment in the ventral areas of the spinal cord. Averaged values for the blood velocity and blood flow indices in each subregion of interest are reported in Supplementary Table S1B. In the ventral horns of the grey matter, a significant decrease between HC and DCM in blood velocity (Δ =  − 20.1%, *p* = 0.0009, HC: 16.7 ± 3.6 × 10^−3^ [mm^2^/s], DCM: 13.4 ± 3.8 × 10^−3^ [mm^2^/s]) and blood flow indices (Δ =  − 28.2%, *p* = 0.0008, HC: 9.4 ± 3.3 × 10^−4^ [mm^2^/s], DCM: 6.8 ± 2.6 × 10^−4^ [mm^2^/s]) was seen (Fig. 5A). There was no significant difference between cohorts in the dorsal horns in terms of blood velocity (HC: 17.5 ± 4.1 × 10^−3^ [mm^2^/s], DCM: 17.0 ± 5.3 × 10^−3^ [mm^2^/s]) or blood flow indices (HC: 6.4 ± 2.3 × 10^−4^ [mm^2^/s], DCM: 6.0 ± 2.5 × 10^−4^ [mm^2^/s]). In the white matter, a significant decrease in blood velocity (*p* = 0.002) and blood flow indices (*p* = 0.009) was observed between DCM and HC (Fig. 5B). Post-hoc analysis showed that the subregions of interest in which the blood velocity index was affected were the ventral funiculi (Δ =  − 18.2%, *p* = 0.01, HC: 19.9 ± 5.5 × 10^−3^ [mm^2^/s], DCM: 16.3 ± 5.8 × 10^−3^ [mm^2^/s]) and lateral funiculi (Δ =  − 8.5%, *p* = 0.03, HC: 22.1 ± 3.7 × 10^−3^ [mm^2^/s], DCM: 20.2 ± 3.6 × 10^−3^ [mm^2^/s]), as for the blood flow index (ventral funiculi: Δ =  − 21.5%, *p* = 0.04, HC: 13.4 ± 6.5 × 10^−4^ [mm^2^/s], DCM: 10.5 ± 5.4 × 10^−4^ [mm^2^/s]; lateral funiculi: Δ =  − 16.5%, *p* = 0.03, HC: 8.2 ± 2.5 × 10^−4^ [mm^2^/s], DCM: 6.9 ± 2.8 × 10^−4^ [mm^2^/s]). No differences were observed between patients and healthy controls in the dorsal columns regarding the blood velocity (HC: 19.0 ± 4.4 × 10^−3^ [mm^2^/s], DCM: 17.5 ± 4.6 × 10^−3^ [mm^2^/s]) and blood flow indices (HC: 6.2 ± 1.8 × 10^−4^ [mm^2^/s], DCM: 5.5 ± 2.3 × 10^−4^ [mm^2^/s]).

### Relationship between remote atrophy and microvascular volume

**Figure 6:**
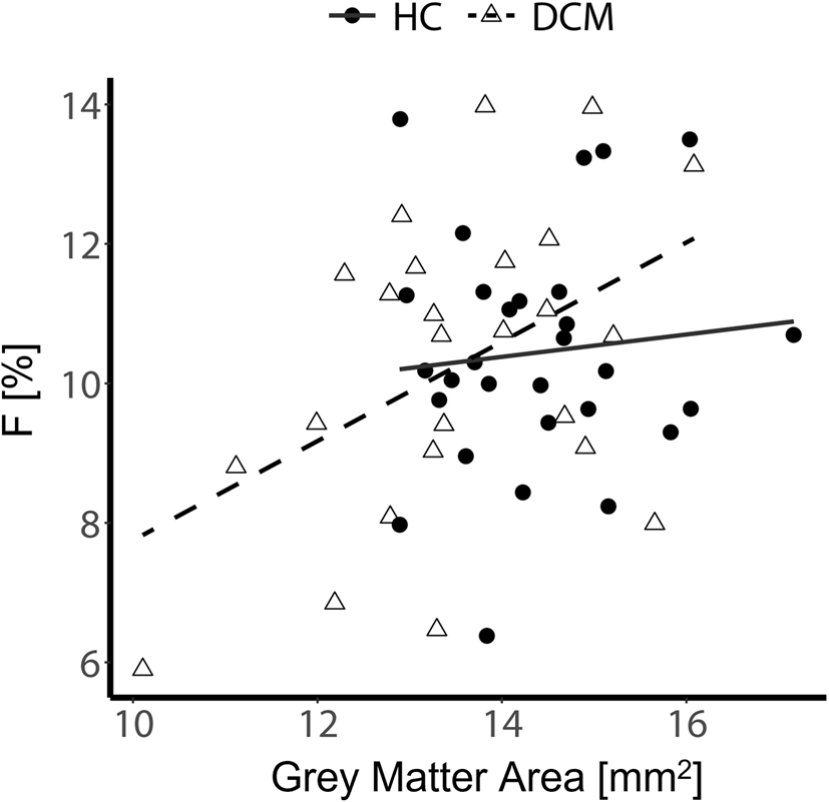
Perfusion deficit and grey matter atrophy. Correlation between microvascular volume fraction and grey matter atrophy in DCM and healthy controls (HC). Linear regression models of the microvascular volume fraction (${\text{F}}$) [%] and grey matter cross-sectional area [mm^2^] averaged across C1-C3 levels in HC and DCM patients.

There was a significant positive correlation between the microvascular volume fraction ($F$) and the grey matter cross-sectional area in DCM patients. While the group interaction (HC ~ DCM) was not significant (*p* = 0.2), post-hoc analysis indicated that the correlation was mainly driven by DCM patients (*R* = 0.46, *p* = 0.02). Moreover, no correlation was observed in the healthy control group (*R* = 0.10, *p* = 0.6) (Fig. 6). A similar trend was observed in the white matter of DCM patients, although not significant (*R* = 0.3, *p* = 0.14).

### Relationship between remote hemodynamic impairment and clinical impairment

No significant associations were observed between perfusion parameters and the collected neurological readouts, nor with the MSCC, MCC, or stenosis level.

### Relationship between remote atrophy and clinical impairment

A significant correlation was observed between the white matter cross-sectional area and the cervical light-touch score in DCM patients (*R* = 0.42, *p* = 0.042).

## Discussion

This study shows remote tissue-specific perfusion impairment in the cervical cord in DCM patients above the compression site (C1-C3 levels). Blood velocity and blood flow indices were reduced in the atrophied cervical grey and white matter above the level of compression. Notably, decreased microvascular volume fraction was linked to the extent of grey matter atrophy in DCM patients, indicating a correlation between rostral micro-perfusion deficit and macrostructural degeneration. The current findings suggest that the hemodynamics and vasculature architecture are adversely affected far beyond the focal site of compression in DCM. Additionally, this work shows that IVIM may provide a reliable assessment of spinal cord hemodynamic impairment.

Remote neurodegenerative changes following cord compression spread above the stenosis level in DCM, due to anterograde and retrograde axonal degeneration, as well as trans-synaptic changes within the grey matter, leading to cord atrophy^21^. We first confirmed previous findings in DCM^5,7,21,42,43^, by showing a significant decrease of grey and white matter cross-sectional area in DCM compared to controls.

The perfusion maps showed a clear distinction between the microvascular volume fraction ($F$) in grey matter and white matter, with a higher microvascular volume fraction in the ventral grey matter in both cohorts. Those findings are in line with microangiography findings of the spinal cord vasculature^44^ and previous reports investigating perfusion of the cervical cord^15,20^. A schematic figure of the vascularization of the spinal cord^45^ is shown in Fig. 3C for visualization purposes. Of note, the ring of high values seen at the edge of the spinal cord on the blood flow index maps can be explained by partial volume effect with the cerebrospinal fluid and registration imprecisions but could also be partly attributed to the pial arterial plexus, a network of surface vessels surrounding the spinal cord^45^ (Fig. 3C).

Moreover, there was a statistically significant decrease in tissue-specific blood velocity ($D^{*}$) (white matter: − 9.5%; grey matter:—11.0%) and blood flow ($F \cdot D^{*}$) (white matter: − 15.9%, grey matter: − 14.4%) in DCM patients above the level of compression. While evidence of reduction in blood flow at the compression site has been shown in experimental studies modeling progressive cervical cord compression^11,12^, our findings indicate that in DCM patients, the impairment in vascular dynamics extends to multiple segments above the stenosis. This suggests that IVIM parameters are sensitive to perfusion impairment in DCM patients and can enhance our understanding of the underlying mechanisms of hemodynamic changes.

The perfusion was mostly affected in the ventro-lateral areas of the cervical cord, with a significant decrease in blood velocity and blood flow indices in the ventral horns of the grey matter, in the ventral funiculi, and lateral funiculi of DCM patients. The ventro-lateral funiculi comprise the spinothalamic tract and the corticospinal tract, which are mainly affected in DCM due to retrograde and anterograde degeneration following canal compression^21^. Thus, the current findings suggest that there may be an association between hemodynamic damage and neurodegenerative processes remote to the compression. Of note, potential hemodynamic deficit in the dorsal horns may remain unrevealed on the IVIM maps due to their lower vascularization^45^, leading to ultimately small changes. The ventral part of the spinal cord is vascularized by arteries arising from the anterior spinal artery (ASA), which runs longitudinally along the spinal cord. Crucially, the diameter of the ASA has been shown to decrease following cord compression^46^, which could indirectly affect the blood dynamics in the ASA and lead to downstream impairment rostral to the compression. Finally, the compression at the anterior area of the spinal cord (resulting for instance from hypertrophy of the posterior longitudinal ligament or displacement of intervertebral disc material^2^) can be more pronounced in DCM compared to the posterior side^8^. This could explain the compression-induced hemodynamic deficit observed in the ventro-lateral areas in this DCM cohort.

The microvascular volume fraction $\left( F \right)$ values in the white matter and grey matter were in line with values previously reported in IVIM investigations of the spinal cord^20^ and the brain^18,19^ in both cohorts. While damage to the microvasculature architecture with an ensuing reduction in the number of vessels has been observed in preclinical models at the compression site^9,10^, the remote microvascular volume fraction was not significantly different between DCM patients and healthy controls in this study. This finding may indicate that the number of vessels remained the same above the level of compression. Nonetheless, possible compensatory mechanisms and adaptable response from the vascular system, such as neovascularization and vascular remodeling, could be contributing to the microvasculature architecture state. Indeed, evidence of those adaptive processes has been shown in cord compression models in animals^47,48^. Likewise, vascular regenerative capacities in DCM patients may be an underlying key factor in the compressed cord, owing to the chronic and progressive characteristics of the pathology^10^.

The IVIM perfusion indices did not significantly differ between DCM patients with and without radiological evidence of myelopathy, showing that hemodynamics was similarly impaired in the cervical cord of both sub-cohorts.

There was a statistically significant association between the decreasing microvascular volume fraction and grey matter atrophy in DCM patients. This correlation was not observed in healthy controls. Thus, there may be a possible alteration in the grey matter microvasculature volume related to the tissue atrophy observed in DCM, indicating possible remote microvasculature impairment in patients with more severe atrophy. There was no significant correlation between the maximum level of stenosis and the degree of perfusion impairment, indicating that the hemodynamic damage may result from a multi-factorial process following the compression. This may include the level of compression, its severity, and the presence of a single or multiple stenosis sites.

Several limitations should be acknowledged in this study. DCM patients were mildly clinically affected and had neurological outcome measures in the upper end of the clinical data range, leading to ceiling effect in the statistical analysis investigating correlation between IVIM indices and clinical parameters. Of note, the statistical results have not been corrected for multiple comparisons. Furthermore, the IVIM model is sensitive to high fluid velocities, such as that of the cerebrospinal fluid encircling the spinal cord. The signal in voxels at the periphery of the spinal cord may therefore be affected by partial volume effect with the pulsative cerebrospinal fluid (e.g., the ring of high values seen on the blood flow index maps), which can lead to bias during the voxel-wise fit. To minimize this from affecting parameter quantification, all regions of interest on the perfusion maps were eroded at the cord periphery. Finally, the current acquisition time of the IVIM protocol hinders its translation to clinical routine and future research focusing on reduction of scan time (e.g., through optimization of the b-value distribution and number of repetitions) is warranted.

In conclusion, cervical cord tissue-specific hemodynamic deficit rostral to the site of compression is evident in DCM patients. Moreover, the correlation between perfusion impairment and grey matter atrophy indicated that compression-induced perfusion changes were associated to the underlying neurodegenerative processes. Thus, IVIM MRI is sensitive to hemodynamic impairment and provides a reliable non-invasive tool to investigate cervical cord perfusion. Future studies following DCM patients serially can shed light on the underlying mechanisms of remote vasculature reorganization occurring spontaneously and how this is influenced by spinal cord decompressive surgery.

### Supplementary Information

Supplementary Table S1.

## Data Availability

The datasets used and analyzed during the current study are available from the corresponding author on reasonable request.
