# Supplementary material for: Investigation of perfusion impairment in degenerative cervical myelopathy beyond the site of cord compression
Source: Sci Rep. 2023 Dec 19;13:22660. doi: 10.1038/s41598-023-49896-3 (PMC10730822; doi:10.1038/s41598-023-49896-3)
Supplement: Supplementary file 1 — Supplementary Table S1. [file 41598_2023_49896_MOESM1_ESM.pdf]

Investigation of perfusion impairment in degenerative cervical myelopathy beyond the site of cord compression

Anna Lebret[1], Simon Lévy[2,3,4], Nikolai Pfender[1], Mazda Farshad[5], Franziska C.S. Altorfer[5], Virginie Callot[2,3], Armin Curt[1], Patrick Freund[1,6,7], and Maryam Seif[1,7]

[1]*Spinal Cord Injury Center, Balgrist University Hospital, Zurich, Switzerland;*

[2]*Aix-Marseille Univ, CNRS, CRMBM, Marseille, France;*

[3]*APHM, Hôpital Universitaire Timone, CEMEREM, Marseille, France;*

[4]*MR Research Collaborations, Siemens Healthcare Pty Ltd, Melbourne, Australia;*

[5]*Department of Orthopedic Surgery, Balgrist University Hospital, Zurich, Switzerland;*

[6]*Department of Brain Repair and Rehabilitation, Wellcome Trust Center for Neuroimaging, Institute of Neurology, University College London, United Kingdom*

[7]*Department of Neurophysics, Max Planck Institute for Human Cognitive and Brain Sciences, Leipzig, Germany*

Corresponding author: Maryam Seif,
Spinal Cord Injury Center,
Balgrist University Hospital,
Forchstrasse 340, 8008 Zürich,
Switzerland.
Email: maryam.seif@balgrist.ch,
Phone: +41 (0) 44 510 72 14.

Supplementary Table S1: Spinal cord tissue-specific IVIM parameters

(A)

	D^* [mm²/s] ×10⁻³		$F \cdot D^*$ [mm²/s] ×10⁻⁴		F [%]		D [mm²/s] ×10⁻⁴	
	WM	*GM*	*WM*	*GM*	*WM*	*GM*	*WM*	*GM*
**HC**	20.1 ± 3.9	14.3 ± 3.1	8.0 ± 2.5	7.5 ± 1.6	6.4 ± 1.5	10.4 ± 1.7	4.0 ± 0.6	4.3 ± 0.6
**DCM**	18.7 ± 3.9	12.7 ± 2.9	6.7 ± 2.7	6.4 ± 1.9	6.3 ± 1.6	10.3 ± 2.1	4.1 ± 0.6	4.4 ± 0.6
**Difference**	-9.5%	-11.0%	-15.9%	-14.4%	-0.3%	-1.5%	3.2%	1.6%
***p* value**	0.03	0.03	0.04	0.02	0.5	0.4	0.8	0.7

(B)

	D^* [mm²/s] ×10⁻³				
	VH	*DH*	*VF*	*LF*	*DC*
**HC**	16.7 ± 3.6	17.5 ± 4.1	19.9 ± 5.5	22.1 ± 3.7	19.0 ± 4.4
**DCM**	13.4 ± 3.8	17.0 ± 5.3	16.3 ± 5.8	20.2 ± 3.6	17.5 ± 4.6
**Difference**	-20.1%	-2.7%	-18.2%	-8.5%	-7.7%
***p* value**	0.0009	0.4	0.01	0.03	0.1

	$F \cdot D^*$ [mm²/s] ×10⁻⁴				
	VH	*DH*	*VF*	*LF*	*DC*
**HC**	9.4 ± 3.3	6.4 ± 2.3	13.4 ± 6.5	8.2 ± 2.5	6.2 ± 1.8
**DCM**	6.8 ± 2.6	6.0 ± 2.5	10.5 ± 5.4	6.9 ± 2.8	5.5 ± 2.3
**Difference**	-28.2%	-7.1%	-21.5%	-16.5%	-10.6%
***p* value**	0.0008	0.2	0.04	0.03	0.1

(A) IVIM parameters (blood velocity index (D^*), blood flow index ($F \cdot D^*$), microvasculature volume fraction (F), and diffusion coefficient (D)) in healthy controls (HC) and DCM patients, averaged across participants within the groups in the white matter (WM) and grey matter (GM). (B) Tissue-specific blood velocity (D^*) and blood flow ($F \cdot D^*$) indices in healthy controls (HC) and DCM patients, averaged across participants within group in the ventral horns (VH), dorsal horns (DH) of the grey matter and in the ventral funiculi (VF), lateral funiculi (LF) and dorsal columns (DC) of the white matter. Differences in percentage between mean values in DCM patients compared to healthy controls, along with the corresponding p-value, are also reported.
